# Recombinant R-spondin2 and Wnt3a Up- and Down-Regulate Novel Target Genes in C57MG Mouse Mammary Epithelial Cells

**DOI:** 10.1371/journal.pone.0029455

**Published:** 2012-01-04

**Authors:** Bolormaa Baljinnyam, Malgorzata Klauzinska, Saad Saffo, Robert Callahan, Jeffrey S. Rubin

**Affiliations:** 1 Laboratory of Cellular and Molecular Biology, National Cancer Institute, Bethesda, Maryland, United States of America; 2 Oncogenetics Section, Mammary Biology and Tumorigenesis Laboratory, National Cancer Institute, Bethesda, Maryland, United States of America; Wayne State University, United States of America

## Abstract

R-spondins (Rspos) comprise a family of four secreted proteins that have important roles in cell proliferation, cell fate determination and organogenesis. Rspos typically exert their effects by potentiating the Wnt/β-catenin signaling pathway. To systematically investigate the impact of Rspo/Wnt on gene expression, we performed a microarray analysis using C57MG mouse mammary epithelial cells treated with recombinant Rspo2 and/or Wnt3a. We observed the up- and down-regulation of several previously unidentified target genes, including ones that encode proteins involved in immune responses, effectors of other growth factor signaling pathways and transcription factors. Dozens of these changes were validated by quantitative real time RT-PCR. Time course experiments showed that Rspo2 typically had little or no effect on Wnt-dependent gene expression at 3 or 6 h, but enhanced expression at 24 h, consistent with biochemical data indicating that Rspo2 acts primarily to sustain rather than acutely increase Wnt pathway activation. Up-regulation of gene expression was inhibited by pre-treatment with Dickkopf1, a Wnt/β-catenin pathway antagonist, and by siRNA knockdown of β-catenin expression. While Dickkopf1 blocked Rspo2/Wnt3a-dependent down-regulation, a number of down-regulated genes were not affected by β-catenin knockdown, suggesting that in these instances down-regulation was mediated by a β-catenin-independent mechanism.

## Introduction

The R-spondin (Rspo) protein family consists of four homologous members that are dynamically expressed during embryogenesis and have important roles in development [1]–[5]. *Rspo2* participates in myogenesis, limb, laryngeal-tracheal and lung morphogenesis, osteoblast maturation and bone mineralization [4], [6]–[9], while *Rspo3* is required for placental formation, angioblast and vascular development [10], [11]. Mutations in particular *Rspo* genes cause human syndromes characterized by specific birth defects. For example, loss-of-function *Rspo1* mutations result in female-to-male gender reversal due to aberrant ovarian development [12], and *Rspo4* point mutations are associated with absent or malformed fingernails and toenails (anonychia) [13], [14].

Several lines of evidence have linked *Rspos* to the *Wnt* genes. The Wnts comprise a large family of secreted, lipid-modified glycoproteins that have a variety of crucial functions during embryonic development and contribute to tissue homeostasis in the adult [15]–[17]. *Rspo* and *Wnt* genes exhibit overlapping patterns of expression in many tissues during development, including the neural tube and muscle [2]–[4]. Together they induce myogenesis by stimulating the transcriptional activity of β-catenin [4], a key mediator of Wnt signaling that promotes differentiation or proliferation depending on the context [16]. Administration of Rspo protein to mice elicited a massive proliferative expansion of the small intestines, consistent with a direct effect on Wnt/β-catenin signaling in the crypts [18]. Recombinant Rspo supports the propagation of intestinal crypt and villus structures in three-dimensional *ex vivo* cultures [19]. Activation of the β-catenin pathway requires Wnt binding to receptors in the Frizzled family and co-receptors, low density lipoprotein receptor-related proteins 5 or 6 (LRP5/6). These interactions trigger phosphorylation of LRP5/6 and disassembly of a β-catenin degradation complex, which allows β-catenin to accumulate in the cytosol and translocate to the nucleus where it functions as a transcriptional co-activator [20], [21]. How Rspos enhance signaling through this pathway is uncertain. Reports vary regarding the ability of Rspos to bind Frizzleds, LRP6 or Wnts [22], [23]. One group suggested that Rspos inhibit LRP6 internalization and down-regulation [24], [25], although others disputed this mechanism [26]–[28]. Recent articles claim that LGR4/5/6 (leucine-rich repeat containing G protein-coupled receptor) function as Rspo receptors and regulate Wnt/β-catenin signaling through association with Wnt receptors [29]–[31].

The connection between Wnt and Rspo extends to cancer. *Wnt1* was identified as a gene that was insertionally activated by the mouse mammary tumor virus (MMTV) [32], [33], and subsequently shown to stimulate the formation of adenocarcinomas when expressed as a transgene in mouse mammary gland [34]. Expression of *Rspo2* and other *Rspo* family members also was induced by insertion of MMTV into mammary epithelial cell DNA, and similarly associated with tumorigenesis [35], [36]. Interestingly, activation of *Rspo* genes in MMTV-derived tumors was accompanied by independent activation of *Wnt* or *Fgf* genes, with *Rspo2* and *Wnt3a* expression being the most frequent combination [35]. Moreover, our recent study suggested that *Rspos* and *Wnts* cooperate to promote malignant transformation and/or influence tumor behavior of mammary cells [37]. Considering the well-established role of Wnt/β-catenin signaling in several types of cancer [15]–[17], stimulation of tumorigenesis via this pathway would not be surprising. However, other Wnt pathways also contribute to neoplasia [38], [39] and Rspo3 was recently shown to activate JNK signaling [40], suggesting that the proposed cooperation of *Rspos* and *Wnts* in cancer might involve other mechanisms as well.

The present study was undertaken to investigate the interaction of Rspo and Wnt in mammary epithelial cells with a primary focus on the regulation of gene expression. We used the C57MG mouse mammary epithelial line as our model system because several earlier reports had characterized Wnt target genes in this setting [41]–[46], facilitating our analysis of Rspo effects on Wnt-dependent gene expression. In contrast to previous studies that relied on the use of crude Wnt-containing conditioned medium or C57MG cells transfected with Wnt cDNA, we used purified recombinant proteins, Rspo2 and Wnt3a, thus ensuring the specificity of elicited responses and enabling time-course and dose-response experiments. Our investigation revealed the up-regulation and down-regulation of many novel Wnt target genes, which often was enhanced by concomitant Rspo2 addition. Typically up-regulation was mediated by β-catenin, as indicated by inhibition with small interfering RNA (siRNA) that blocked β-catenin expression and the Wnt/β-catenin pathway antagonist Dickkopf 1 (Dkk1) [47]. Interestingly, Dkk1 also inhibited the down-regulation of Wnt3a/Rspo2 target genes, but only a subset of these decreases in expression was disrupted by knockdown of β-catenin. This suggested that β-catenin-independent mechanisms elicited by Wnt3a and Rspo2 also control gene expression in mammary epithelial cells.

## Results and Discussion

### Microarray analysis of Rspo2/Wnt3a-dependent gene expression in C57MG cells

**Figure 1:**
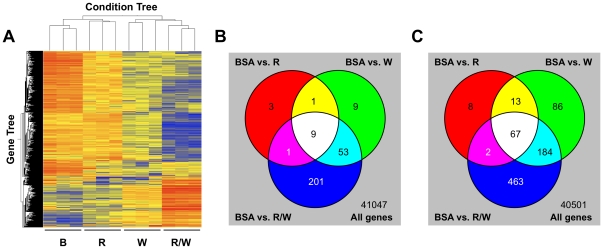
Schematic summary of Rspo2/Wnt3a-dependent gene expression in C57MG cells. (**A**) Heat map shows genes that were up-regulated (red) or down-regulated (blue) at least two-fold relative to the normal value (yellow). Microarray analysis was performed with RNA harvested and processed individually from three monolayers for each treatment group [B = BSA (16 ug/ml); R = Rspo2 (100 ng/ml); W = Wnt3a (100 ng/ml); R/W = Rspo2 (100 ng/ml) + Wnt3a (100 ng/ml)]. (**B**) Venn diagram indicates the number of genes that were up-regulated uniquely or in common by Rspo2, Wnt3a or the combination of both. (**C**) Venn diagram indicates the number of genes that were down-regulated uniquely or in common by Rspo2, Wnt3a or the combination of both.

Initially we monitored the expression of prototypical Wnt target genes (*Axin2*, *Ccnd1*, *Msln*; for accession numbers of genes mentioned in this article see Table S2) by quantitative real-time RT-PCR (qRT-PCR) in time-course and dose-response experiments to optimize conditions for the microarray analysis. Based on these results, C57MG cells were incubated for 24 h with 100 ng/ml Rspo2 or Wnt3a alone or in combination, or with the carrier protein bovine serum albumin (BSA, 16 µg/ml). Total RNA was harvested from triplicate monolayers corresponding to each treatment group and analyzed with Agilent's Whole Mouse Genome Oligo-4×44K microarray, which is comprised of 41,534 60-mer oligonucleotide probes representing over 41,000 transcripts. A total of 22,696 transcripts were detected in the C57MG cells. Expression of 1100 genes was altered at least two-fold by Rspo2 and Wnt3a alone or together, relative to the BSA control (p<0.05). Distinct patterns of expression were associated with each treatment group as illustrated by a heat map of differentially expressed genes (Figure 1A). The greatest number of changes in expression was observed with the combination of Rspo2 and Wnt3a. Not only were genes up-regulated by Rspo2 plus Wnt3a (Figure 1A, lower portion of heat map), but an even larger number were down-regulated by this treatment (Figure 1A, upper and middle portion). Approximately 25% of the affected genes were up-regulated (Figure 1B) and 75% down-regulated (Figure 1C) by the recombinant proteins. Rspo2 alone altered the expression of relatively few genes (14 up-regulated, 90 down-regulated), and most (90) were affected by Wnt3a as well (Figure 1B and 1C). While Wnt3a increased the expression of 72 genes and decreased the expression of 350, an additional 664 transcripts were only altered by the combination of Rspo2 and Wnt3a (Figure 1B and 1C). These results reinforced the idea that Rspo2 and Wnt3a have overlapping and synergistic effects on gene expression.

Characterization of Rspo2/Wnt3a responsive genes according to gene ontology classification showed a strong correlation with development (183 genes, P-value 2.06E-09) and signal transduction (211 genes, P-value 1.08E-06). This was not surprising given the established association of Rspos and Wnts with these biological processes. However, the diversity of subcategories within these groups was noteworthy, implying potential relevance beyond mammary cell biology and extensive crosstalk with other signaling pathways (Table S1). Unexpectedly, there was an even stronger link to genes involved in immune response (89 genes, P-value 1.15E-19), including innate immunity and inflammation (Table S1). While contributions of Wnt signaling to the development and function of the immune system have been described [48], [49], many of the immune response genes detected in our microarray analysis were not previously associated with Wnt or Rspo regulation and therefore suggest novel mechanisms of immune regulation. In addition to these gene ontology categories, several putative Rspo2/Wnt target genes were classified among various metabolic processes (Table S1). Interestingly, Wnt/β-catenin signaling was recently reported to control the hepatic expression of genes that mediate gluconeogenesis [50].

### Validation of known and novel target genes

We verified the expression pattern of several dozen genes by qRT-PCR (Table S2), confirming the reliability of the microarray analysis. In a few instances the magnitude of change in gene expression elicited by Rspo2 and/or Wnt3a relative to BSA differed in qRT-PCR vs. microarray analysis, but the overall pattern was similar in a large majority of the genes examined. We observed the up-regulation of several previously identified Wnt/β-catenin target genes including those encoding *Axin2*, *Ccnd1*, *Msln*, *Ctgf*, *Efnb2* and *Twist1* (Table 1). While the expression of these genes is mediated by β-catenin/TCF complexes, we also observed changes that involved β-catenin interactions with other transcription factors such as the retinoic acid receptor-dependent, up-regulation of *Enpp2/autotaxin* [51]. Of note, many of the changes in gene expression confirmed in C57MG cells also were verified by qRT-PCR in another mouse mammary epithelial cell line, NOG-8 (data not shown). Because the regulation of several previously documented Wnt target genes was common to cells from other organs, we suspect that at least some of the new data obtained in the present microarray analysis are likely to be relevant to other tissues and cell types. Interestingly, Rspo2 strongly enhanced the β-catenin-dependent activity of Wnt4 in C57MG cells, which by itself was low compared to Wnt3a. However, a combination of Rspo2 and Wnt5a was unable to stimulate *Axin2* expression in C57MG cells, consistent with the general lack of Wnt5a activity in the β-catenin pathway.

**Table 1:** Microarray data of known Wnt/β-catenin target genes validated by qRT-PCR.

	Rspo2		Wnt3a		Wnt3a/Rspo2	
Gene	Fold change	*P* value	Fold change	*P* value	Fold change	*P* value
*Axin2*	0.75	0.44	1.53	0.17	21.62	0.01
*Rhou*	0.99	0.96	2.22	0.01	12.78	0.15E-2
*Enpp2*	0.75	0.19	1.92	0.02	9.21	0.95E-3
*Ahr*	1.02	0.82	2.1	0.67E-3	7.24	0.90E-3
*Klf5*	1.45	0.01	2.23	0.34E-2	5.06	0.76E-2
*Edn1*	1.41	0.01	2.18	0.19E-2	4.22	0.19E-5
*Ccnd1*	1.39	0.08	2.12	0.02	3.28	0.02
*Msln*	1.14	0.03	1.82	0.39E-2	2.91	0.23E-4
*Ctgf*	1.24	0.06	1.84	0.11E-2	2.52	0.67E-3
*Efnb2*	1.47	0.02	1.82	0.03	2.3	0.13E-2
*Twist1*	1.19	0.02	1.27	0.09	2.04	0.015

Fold change relative to level in cells treated with BSA.

Among the immune response genes confirmed by qRT-PCR (Table 2), the increase in expression of the chemokine receptor *CXCR6* was of particular interest because of its association with mammary and prostate cancer [52]–[55]. Lin et al. reported that *CXCR6* expression is mediated by HIF1α and contributes to breast cancer metastasis [55]. HIF1α is another transcription factor that acts in concert with β-catenin to regulate a set of target genes [56]; perhaps such cooperation controls *CXCR6* expression as well. The striking increase in *interleukin 33* (*IL33*) also was noteworthy. Although originally associated with T cell activation, IL33 subsequently has been shown to act on a variety of cell types [57]. In contrast to *CXCR6* and *IL33*, the chemokines *CCL5* and *CCL7* were down-regulated by Wnt3a alone and in combination with Rspo2. Both Rspo2 and Wnt3a inhibited the expression of several acute phase reactants (*Saa3, C3, Hp and Lcn2*), in some instances by >90%. While some changes in expression of immune response genes such as *CXCR6* presumably have a direct effect on mammary epithelial cells, others might regulate immune function in the microenvironment. Alternatively, *Saa3* expressed by the bovine mammary gland is transmitted in milk and thought to facilitate immune function in the offspring [58].

**Table 2:** Microarray data of immune response genes validated by qRT-PCR.

	Rspo2		Wnt3a		Wnt3a/Rspo2	
Gene	Fold change	*P* value	Fold change	*P* value	Fold change	*P* value
*IL33*	5.11	0.19E-2	11.03	0.22E-2	33.54	0.36E-4
*CXCR6*	0.74	0.65	2.96	0.04	22.84	0.67E-5
*TGFb2*	1.11	0.39	1.62	0.09	4.38	0.05
*InhbA*	1.40	0.06	2.00	0.52E-3	3.74	0.01
*TGFbR2*	1.03	0.77	1.89	0.05	3.20	0.03
*CCL5*	0.69	0.05	0.58	0.21E-2	0.38	0.39E-3
*IRF7*	0.71	0.32E-3	0.40	0.14E-3	0.28	0.11E-2
*CCL7*	0.40	0.12E-2	0.31	0.15E-2	0.13	0.11E-2
*Saa3*	0.08	0.18E-2	0.09	0.75E-3	0.11	0.20E-3
*C3*	0.22	0.10E-3	0.12	0.39E-3	0.06	0.40E-3
*Hp*	0.16	0.11E-2	0.02	0.53E-2	0.03	0.51E-2
*Lcn2*	0.05	0.31E-2	0.02	0.29E-2	0.01	0.29E-2

Fold change relative to level in cells treated with BSA.

Potential crosstalk between Wnt/Rspo and other growth factor signaling mechanisms was implicit in the synergistic up-regulation of multiple genes that participate in insulin/IGF, TGFβ and FGF pathways (Table 3). One of these genes, *IRS1*, also was identified as a pathophysiologically relevant Wnt/β-catenin target gene in colorectal carcinomas [59]. Up-regulation of insulin/IGF pathway genes, including *IGF1*, is provocative given the association of IGF signaling and breast cancer [60]. Numerous studies have described interactions between Wnt and TGFβ or Wnt and FGF signaling [61], [62]. The present data extend these observations by suggesting that Wnt/Rspo signaling regulates the expression of various effectors in these growth factor pathways.

**Table 3:** Microarray data of genes in other growth factor signaling pathways.

	Rspo2		Wnt3a		Wnt3a/Rspo2	
Gene	Fold change	*P* value	Fold change	*P* value	Fold change	*P* value
*IGFbp2*	0.99	0.98	2.64	0.02	8.82	0.57E-2
*TGFb2*	1.11	0.39	1.62	0.09	4.38	0.05
*Spry1*	1.64	0.49E-2	2.42	0.21E-3	4.20	0.42E-4
*IGF1*	1.31	0.28	2.04	0.53E-2	3.21	0.81E-2
*TGFbR2*	1.03	0.77	1.89	0.05	3.20	0.03
*IRS1*	1.08	0.53	1.71	0.67E-2	3.06	0.17E-2
*FGFR5*	1.08	0.27	1.17	0.45	2.47	0.54E-2
*FGF21*	1.43	0.32	2.35	0.48E-2	2.37	0.07

Fold change relative to level in cells treated with BSA.

We verified that Rspo2 and/or Wnt3a controlled the expression of several transcription factors (Table 4). *Klf5* had been identified as a Wnt target gene in multiple settings including C57MG cells [45], [46], while the up-regulation of *Helt* was a novel finding as was the down-regulation of *Klf15*, *IRF7* and *Stat1* (Table 4). Many other confirmed, previously unrecognized Wnt target genes are listed in Table 5. Included in this group are genes encoding the tyrosine kinases HCK and Tec, angiopoietin1 and angiopoietin-like4, and inscuteable, a mediator of cell polarity.

**Table 4:** Microarray data of transcription factor genes validated by qRT-PCR.

	Rspo2		Wnt3a		Wnt3a/Rspo2	
Gene	Fold change	*P* value	Fold change	*P* value	Fold change	*P* value
*Helt*	1.48	0.97	2.97	0.31E-2	7.80	0.16E-2
*Ahr*	1.02	0.82	2.10	0.67E-3	7.24	0.89E-3
*Klf5*	1.45	0.01	2.23	0.34E-2	5.06	0.76E-2
*TCF4*	1.29	0.59E-2	1.39	0.01	2.20	0.01
*Nanog*	2.05	0.73E-2	1.88	0.02	2.18	0.45E-2
*Klf15*	0.50	0.01	0.43	0.33E-2	0.30	0.39E-3
*IRF7*	0.71	0.32E-3	0.40	0.14E-3	0.28	0.11E-2
*Stat1*	0.50	0.04	0.31	0.02	0.22	0.02

Fold change relative to level in cells treated with BSA.

**Table 5:** Microarray data of additional novel target genes validated by qRT-PCR.

	Rspo2		Wnt3a		Wnt3a/Rspo2	
Gene	Fold change	*P* value	Fold change	*P* value	Fold change	*P* value
*PpFibp2*	1.52	0.07	4.30	0.88E-2	16.34	0.12E-2
*HCK*	1.13	0.79	3.49	0.02	13.46	0.97E-2
*Tspan18*	0.90	0.77	1.56	0.16	12.19	0.27E-4
*Tec*	0.93	0.68	2.68	0.03	11.54	0.29E-2
*Chrna1*	1.32	0.42	1.94	0.13	6.29	0.79E-2
*Klhl30*	1.66	0.17	2.23	0.43E-3	6.13	0.41E-5
*Insc*	1.55	0.13E-2	2.79	0.35E-3	5.64	0.12E-2
*Epac1*	0.92	0.26	2.71	0.01	4.83	0.12E-5
*Itgbl1*	2.15	0.06	2.59	0.91E-2	4.45	0.54E-3
*FHL1*	1.34	0.09	2.48	0.01	3.26	0.31E-2
*Wnt9a*	1.16	0.52	1.48	0.15	2.97	0.21E-2
*Rnd3*	1.15	0.39E-2	1.33	0.02	2.33	0.21E-2
*Sox9*	1.14	0.19	0.66	0.08	0.42	0.31E-2
*Angptl4*	0.43	0.04	0.25	0.01	0.33	0.01
*Smoc1*	0.97	0.76	0.59	0.03	0.26	0.34E-2
*Angpt1*	0.89	0.18	0.42	0.80E-3	0.17	0.17E-2

Fold change relative to level in cells treated with BSA.

Induction of the aryl hydrocarbon receptor (*Ahr*) by Wnt3a and Rspo2 was especially noteworthy. Though described as a Wnt target gene in a few prostate carcinoma lines [63], this relationship had not been noted elsewhere. Ahr was first identified as the dioxane receptor and associated with its carcinogenic and other toxic effects. Subsequently Ahr was recognized as a transcription factor that bound endogenous or xenobiotic ligands and heterodimerized with Arnt/HIF2 to activate the expression of genes such as Cyp1a1 [64]. More recently Ahr was shown to function as an E3 ubiquitin ligase that targeted the estrogen and androgen receptors for degradation [65], [66], an activity that might be exploited in cancer therapy [67], [68]. Remarkably, Ahr also serves as an E3 ubiquitin ligase for β-catenin and treatment with natural Ahr ligands suppressed intestinal carcinogenesis in Apc^Min/+^ mice [69]. This implied that the up-regulation of Ahr could provide a mechanism to suppress tumorigenesis by administering Ahr ligands. Such an approach might be useful for the treatment and/or chemoprevention of colorectal, breast and perhaps other cancers. Conversely, Ahr ligand stimulated the expression of *Rspo1* in a zebrafish model of regenerating fin, and thereby enhanced Wnt/β-catenin signaling [70]. Other recent articles have described an interplay of Ahr and Wnt/β-catenin signaling that results in either increase or decrease in target gene expression [71], [72]. It appears that the ability of Ahr to promote or impede β-catenin transcriptional activity is context-specific.

### Rspo2 promotes Wnt3a-dependent gene expression by prolonging β-catenin pathway activation

**Figure 2:**
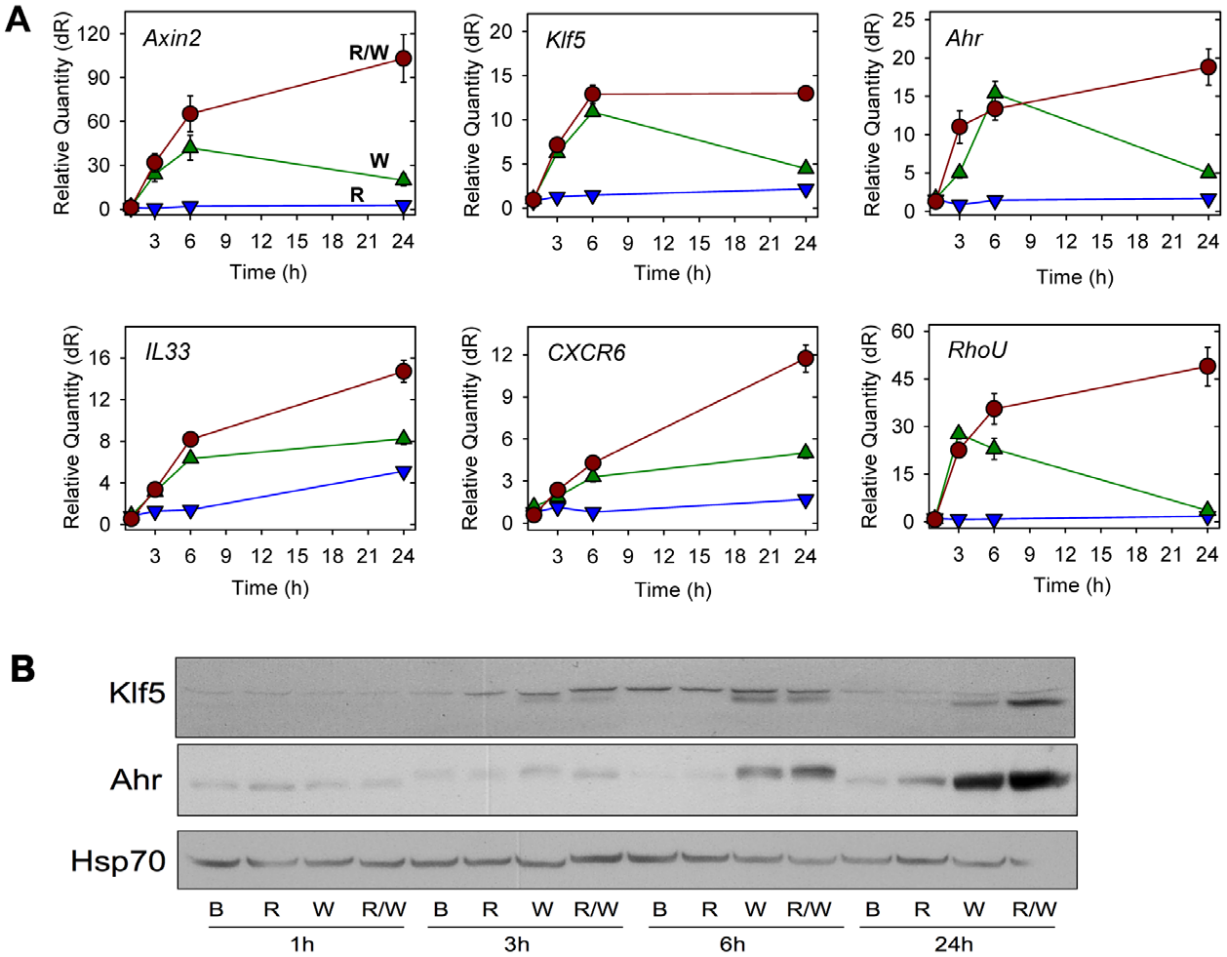
Time-course of Rspo2- and Wnt3a-dependent gene up-regulation in C57MG cells. (**A**) Quantitative RT-PCR analysis of specific gene expression in response to Rspo2 (100 ng/ml) alone (blue), Wnt3a (100 ng/ml) alone (green) or the two together (red) at the indicated time points. Data are the means +/− S.D. (error bars, sometimes too small to see) of triplicate measurements from one of several experiments with similar results. (**B**) Immunoblot analysis of Klf5 and Ahr protein expression following treatment with BSA carrier, Rspo2 (100 ng/ml), Wnt3a (100 ng/ml) alone or in combination for the indicated periods. Membranes were re-probed for Hsp70 as a loading control.

**Figure 3:**
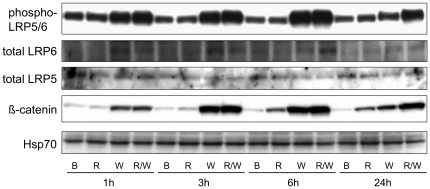
Time-course of Rspo2- and Wnt3a-dependent LRP5/6 phosphorylation and β-catenin stabilization. C57MG cells were treated with BSA, Rspo2 (100 ng/ml), Wnt3a (100 ng/ml) alone or in combination for the indicated periods. Cell lysates (30 µg protein/lane) were immunoblotted for phosphorylated LRP5/6, total LRP5, total LRP6 and Hsp70, the latter serving as a loading control. Soluble β-catenin was measured by immunoblotting after pull-down with GST-E-cadherin.

Time-course experiments with recombinant Wnt3a indicated that gene expression typically was increased at 3 h, reached a peak at 6 h and was reduced at 24 h (Figure 2A). Rspo2 had minor effects on its own or with Wnt3a at 3 and 6 h, but it markedly enhanced expression of transcript at 24 h (Figure 2A). Though stimulation was not as dramatic at the protein level, immunoblot analysis confirmed the up-regulation of Klf5 and Ahr, with the effect of Rspo2 evident at 24 h (Figure 2B). The doublet bands in the Klf5 blot probably reflected differential post-translational modification, which had been previously described [73]. Similar observations about the timing of responses to Rspo2 and Wnt3a were made with standard biochemical readouts of β-catenin pathway activation. Rspo2 alone elicited only a small increase in phosphorylated LRP5/6 and the accumulation of β-catenin protein (Figure 3). However, Rspo2 enhanced the activity of Wnt3a in these assays at 24 h (Figure 3). These results demonstrated that the addition of Rspo2 to Wnt3a caused a more sustained activation of the β-catenin pathway, reinforcing our findings with HEK293/STF cells and a truncated Rspo2 derivative [28].

**Figure 4:**
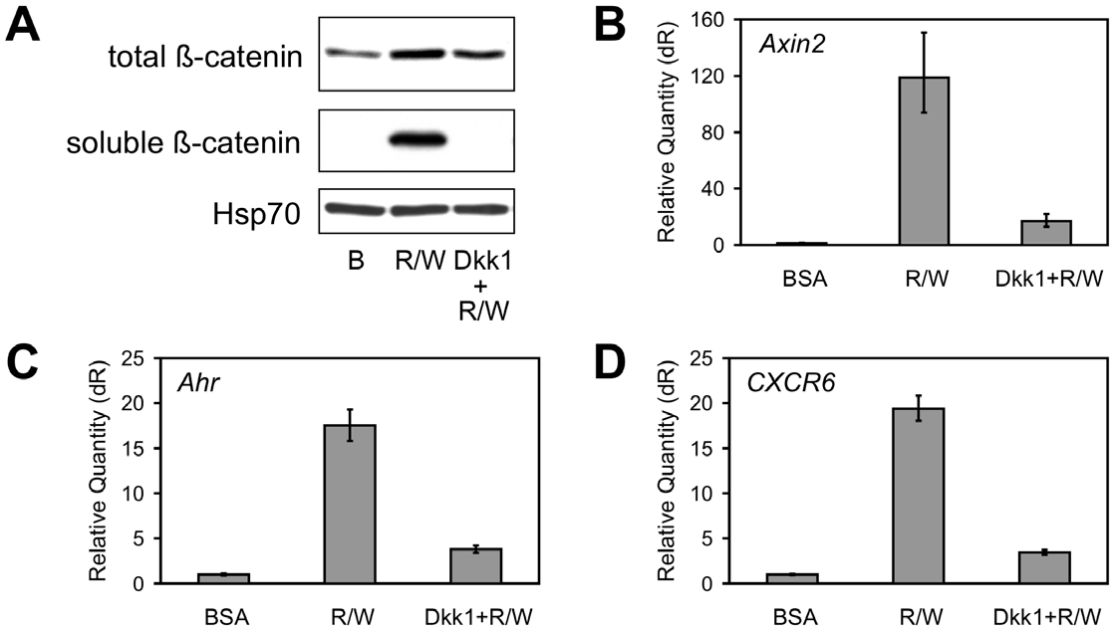
Dkk1 inhibited up-regulation of genes by Rspo2 and Wnt3a. (**A**) Immunoblot analysis of total β-catenin (cell lysate, 30 µg protein/lane) and soluble β-catenin (GST-E-cadherin pull-down) from C57MG cells incubated for 3 h with BSA or Rspo2 (10 ng/ml) plus Wnt3a (100 ng/ml), with or without 20 min Dkk1 (1 µg/ml) pre-treatment. Western blot of Hsp70 was loading control. (**B–D**) Quantitative PCR analysis of *Axin2*, *Ahr* and *CXCR6* expression by C57MG cells after 24 h incubation with BSA or Rspo2 plus Wnt3a, with or without Dkk1 pre-treatment [experiment performed in parallel with (A), using the same reagent concentrations]. Results are means +/− S.D. of triplicate measurements from one of four representative experiments.

**Figure 5:**
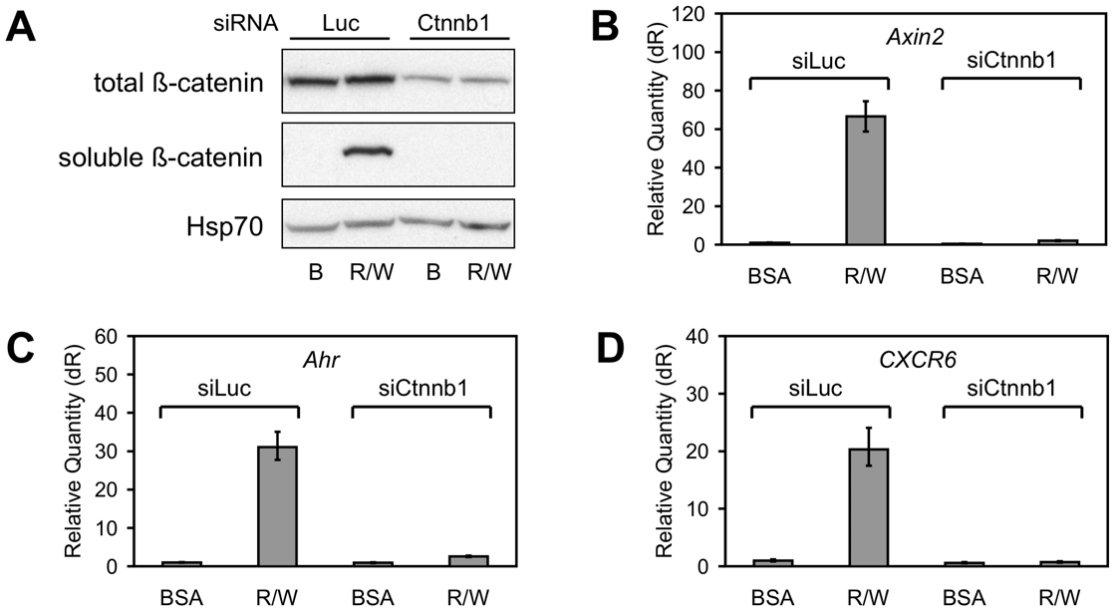
Knockdown of β-catenin inhibited up-regulation of genes by Rspo2 and Wnt3a. (**A**) C57MG cells were pre-treated with either luciferase or pooled Ctnnb1 siRNA reagent for 24 h prior to additional 24 h incubation with BSA or Rspo2 (10 ng/ml) plus Wnt3a (100 ng/ml). Total and soluble β-catenin and Hsp70 were detected as described in Fig. 4 legend. (**B–D**) Quantitative PCR analysis of *Axin2*, *Ahr* and *CXCR6* expression by C57MG cells after 24 h incubation with luciferase or pooled Ctnnb1 siRNA reagent followed by 24 h treatment with BSA or Rspo2 plus Wnt3a [experiment performed in parallel with (A), using the same reagent concentrations]. Results are means +/− S.D. of triplicate measurements from one of three representative experiments.

Proof that the β-catenin pathway was responsible for the up-regulation of validated target genes came from experiments involving Dkk1, an antagonist of this pathway, and small interfering RNA knockdown of *Ctnnb1* (siCtnnb1), the gene encoding β-catenin. Pre-treatment of C57MG cells with recombinant Dkk1 inhibited the stabilization of β-catenin protein (Figure 4A) and blocked the up-regulation of genes induced by the combination of Rspo2 (10 ng/ml) and Wnt3a (100 ng/ml) (Figure 4B–4D and Table S3). Targeting expression with siCtnnb1 decreased the amount of β-catenin protein in the absence of growth factor and blocked its accumulation in response to Rspo2 plus Wnt3a (Figure 5A). As with Dkk1, β-catenin knockdown disrupted the induction of *Axin2*, *Ahr* and *CXCR6* mRNA (Figure 5B–5D) as well as other gene transcripts (Table S4). Similar results were obtained with two independent siCtnnb1 reagents (data not shown). Taken together, these data established that the up-regulation of Rspo2/Wnt3a target genes was mediated by the β-catenin pathway and Rspo2 potentiated expression by extending the duration of pathway activation.

### Down-regulation of genes by Wnt3a is rapid, Dkk1-sensitive and occurs via either a β-catenin-dependent or -independent mechanism

**Figure 6:**
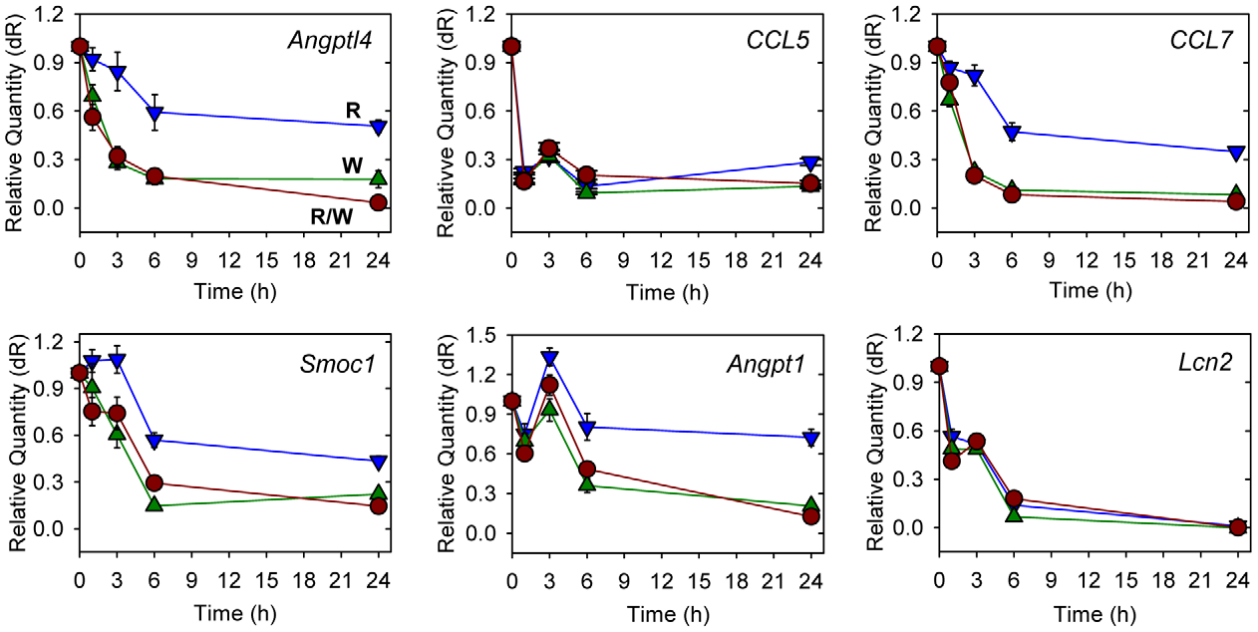
Time-course of Rspo2- and Wnt3a-dependent gene down-regulation in C57MG cells. Quantitative PCR analysis of specific gene expression in response to Rspo2 (100 ng/ml) alone (blue), Wnt3a (100 ng/ml) alone (green) or the two together (red) at the indicated time points. Data are the means +/− S.D. (error bars, sometimes too small to be seen) of triplicate measurements from one of several experiments with similar results.

**Figure 7:**
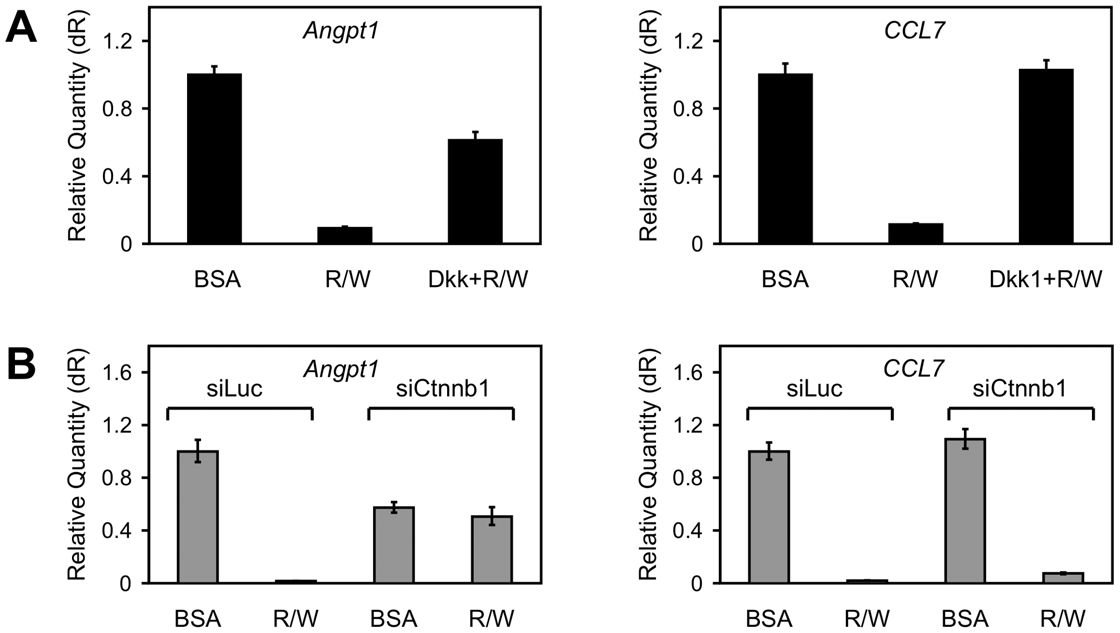
Contrasting effects of Dkk1 and β-catenin knockdown on representative genes down-regulated by Rspo2 and Wnt3a. (**A**) Quantitative RT-PCR analysis of *Angpt1* and *CCL7* expression following 24 h incubation with BSA or Rspo2 (10 ng/ml) plus Wnt3a (100 ng/ml), with or without Dkk1 (1 µg/ml) pre-treatment. (**B**) Quantitative RT-PCR analysis of *Angpt1* and *CCL7* expression following 24 h incubation with BSA or Rspo2 (10 ng/ml) plus Wnt3a (100 ng/ml) after pre-treatment with siLuc vs. siCtnnb1. Results in (A) and (B) are means +/− S.D. of triplicate measurements from one of three representative experiments performed simultaneously with experiments illustrated in Figures 4 and 5.

Time-course analysis of genes down-regulated by Wnt3a and Rspo2 showed that responses were evident at 3 h and sometimes even at 1 h, with maximal or near maximal inhibition seen at 6 h (Figure 6). In general, the effect of Rspo2 plus Wnt3a was not much stronger than that of Wnt3a alone, although Rspo2 alone often had significant inhibitory activity. It is possible that the inhibitory effect of Rspo2 was dependent on low levels of endogenous Wnt expression, while Wnt3a at 100 ng/ml was sufficient for a near-maximal effect. As with the up-regulated genes, pre-treatment of C57MG cells with Dkk1 blocked much if not all of the Rspo2/Wnt3a activity in the qRT-PCR analysis (Figure 7A and Table S3). However, β-catenin knockdown only disrupted the down-regulation of two genes analyzed, *Angpt1* and *Smoc1*, while having little effect on the others (Figure 7B and Table S4). Knockdown of β-catenin showed similar effects in cells treated with Rspo2 or Wnt3a alone (data not shown). These results implied that β-catenin was not involved in the down-regulation of most genes identified as Rspo2/Wnt targets in this study.

Typically Wnt-dependent, β-catenin-mediated gene expression has been associated with stimulation of gene expression. These effects require interaction with other transcription factors such as the DNA-binding TCF family members, as well as retinoic acid receptor, vitamin D receptor or HIF1α [15]–[17], [42], [51], [56], [74]. Relatively little is known about the mechanisms responsible for the down-regulation of gene expression by Wnts and/or β-catenin. There is one report indicating that Wnt/β-catenin suppression of N-Acetylglucosaminyltransferase III expression is associated with TCF binding sites in the promoter region [75].

Particularly intriguing was the observation that most of the decreases in gene expression induced by Rspo2/Wnt3a were Dkk1-sensitive and yet β-catenin-independent. While we cannot exclude the possibility that residual low levels of β-catenin following siCtnnb1 treatment had participated in the inhibitory process, these results suggest that Dkk1 might function in a manner that does not involve a decline in β-catenin protein levels. We were unable to ascertain whether LRP5/6 were required for the de-repression of gene expression by Dkk1 because repeated efforts to simultaneously knockdown expression of LRP5/6 in C57MG cells were unsuccessful. Thus, the present results raise the possibility that the effects of Dkk1 on the suppression of gene expression by Rspo2/Wnt3a might occur by a novel mechanism distinct from disruption of the canonical Wnt/β-catenin pathway. Elucidation of the mechanisms responsible for Rspo2/Wnt3a-dependent decreases in gene expression and the manner in which the decreases were blocked by Dkk1 should be provocative areas for future research.

## Materials and Methods

### Reagents

Recombinant mouse Wnt3a (cat. no. 1324-WN), human R-spondin2 (cat. no. 3266-RS), mouse Dkk1 (cat. no. 5897-DK), mouse Wnt4 (cat. No. 475-WN) and mouse Wnt5a (cat. No. 645-WN) were purchased from R&D Systems (Minneapolis, MN). Anti-β-catenin antibody (cat. no. 610154) was from BD Biosciences (San Jose, CA). Anti-LRP6 (cat. no. 2560) and anti-phospho-LRP6 (Ser1490; also recognizes pLRP5) (cat. no. 2568) antibodies were from Cell Signaling Technology (Danvers, MA). Anti-LRP5 (cat. no. 365400) was from Invitrogen (Carlsbad, CA). Anti-Klf5 (cat. no. ab24331) and Anti-Ahr (cat. no. ab2770) were purchased from Abcam (Cambridge, MA). Antibody against heat shock protein-70 (Hsp70, sc-7298) was from Santa Cruz Biotechnology (Santa Cruz, CA).

### Cell culture

C57MG mouse mammary epithelial cells [41], a gift from the Varmus lab, were cultured in DMEM (cat. no. 11995, Invitrogen) supplemented with 10% fetal bovine serum and 10 µg/ml insulin (cat. no. 12585-014, Invitrogen) and incubated in 5% CO_2_ at 37°C.

### Microarray analysis

C57MG cells were seeded in 6-well plates (2.5×10^5^ cells/well). After an overnight incubation growth medium was replaced with DMEM lacking supplements, and cells were treated for 24 h with recombinant mouse Wnt3a (100 ng/ml) or recombinant human Rspo2 (100 ng/ml) alone or in combination. For the vehicle control, cells were treated with BSA (16 µg/ml) (cat. no. 81-068-3, Millipore, Kankakee, IL). Each treatment group was prepared in triplicate. Cells were harvested in PBS and snap-frozen on dry ice.

RNA isolation, microarray data generation and data analysis were performed by GenUs Biosystems, Northbrook IL. Total RNA was extracted using Ribopure RNA isolation kit from Ambion (Invitrogen). RNA integrity and quality were assessed using an Agilent Bioanalyzer G2938B with the RNA6000 Nano Lab Chip (Agilent Technologies, Santa Clara, CA). Alexa-555-labeled cRNA was prepared by linear amplification of the poly(A)+ RNA population within the total RNA sample. Briefly, 1 µg of total RNA was reverse transcribed after priming with a DNA oligonucleotide containing the T7 RNA polymerase promoter 5′ to a d(T)24 sequence. After second-strand cDNA synthesis and purification of double-stranded cDNA, in vitro transcription was performed using T7 RNA polymerase. The quantity and quality of the cRNA was determined by spectrophotometry and with an Agilent 2100 Bioanalyzer. One µg of purified cRNA was fragmented to uniform size and applied to Mouse Whole Genome 4×44 k arrays (Design ID no. 014868; Agilent Technologies) in hybridization buffer. Arrays were hybridized at 37°C for 18 h in a rotating incubator, washed, and scanned with a G2565 Microarray Scanner (Agilent Technologies). Arrays were processed with Agilent Feature Extraction v9.5.3.1 software and data were analyzed with GeneSpring GX v7.3.1 software (both Agilent Technologies). Raw and normalized data can be accessed in Gene Expression Omnibus, consistent with MIAME guidelines (www.ncbi.nlm.nih.gov/geo/; accession number GSE32096). To compare individual expression values across arrays, raw intensity data from each gene was normalized to the 75^th^ percentile intensity of each array. Only genes with values greater than background intensity in all replicates of at least one condition were used for further analysis. Volcano plots were used to filter for differentially expressed genes >2 fold and p-values<0.05. P-values were calculated using a two-sample T-test assuming unequal variance.

### Quantitative real-time RT-PCR (qRT-PCR)

Cells were seeded in 6-well plates (2.5×10^5^ or 5×10^5^ cells per well) and incubated for 24 h. Growth medium was replaced with DMEM lacking supplements 30 min prior to treatment with BSA (16 µg/ml), Wnt3a (100 ng/ml), Rspo2 (100 ng/ml) or Rspo2 (10 or 100 ng/ml) and Wnt3a (100 ng/ml) for the indicated times. In experiments involving Dkk1, cells were incubated with Dkk1 (1 µg/ml) for 20 min at 37°C prior to the addition of other proteins.

Total RNA was isolated with RNeasy kit (Qiagen) and treated with DNase I (cat. no. 18068-015, Invitrogen) for 15 min at room temperature followed by incubation with 2.5 mM EDTA for 10 min at 65°C to eliminate contamination by genomic DNA. Co-application reverse transcription was carried out as described previously [76] with the SuperScript III First-Strand Synthesis System for RT-PCR (cat. no. 18080-051, Invitrogen). Primers for qRT-PCR (Table S5) were designed using Primer3 software (http://frodo.wi.mit.edu/primer3/). The specificity of each primer pair was analyzed by agarose gel elecrophoresis and confirmed by a single band with the appropriate size. The qRT-PCR was performed with Brilliant SYBR Green QPCR Master Mix (cat. no. 600548, Agilent Technologies). For each reaction 80 ng cDNA and 2 µl of 10 µM primer set were combined with 2× master mix to a final volume of 20 µl. The reactions were analyzed in triplicate using the Mx3005P QPCR machine (Agilent Technologies) with the following settings: 10 min incubation at 95°C followed by a three-step cycling program with 40 cycles of 30 sec at 95°C, 60 sec at 60°C and 30 sec at 72°C. To verify the specificity of the reaction a dissociation program was run following the manufacturer's protocol. Quantification of 18S-RNA and/or β-actin in each cDNA sample was done to normalize the results. Data analysis was performed with MxPro software.

### siRNA transfection

C57MG cells were transfected with the Neon Transfection System (Invitrogen) according to the manufacturer's protocol. Briefly, 10^6^ cells were placed in 100 µl resuspension buffer and combined with 200 pmol siRNA. Double-stranded short interfering RNAs targeting mouse β-catenin (cat. no. GS12387) were purchased from Qiagen (Valencia, CA). In some experiments we used individual siRNA reagents from this pool (cat. no SI00942039 and SI00942046). Luciferase (Luc) siRNA (target sequence: CGUACGCGGAAUACUUCGA) was synthesized by Dharmacon (Thermo Scientific). Electroporation was carried out with two pulses having a width range of 20 ms and 1400 V. After transfection cells were resuspended in 500 µl growth medium and divided into three wells of a 6-well-plate, each containing 2 ml growth medium. Then cells were cultured for 24 h, followed by addition of recombinant proteins and incubation for another 24 h.

### Immunoblotting

Treatment conditions were the same as described above (see RNA isolation and reverse transcription). After incubation with recombinant proteins cells were washed twice with phosphate buffered saline (PBS) and lysed with buffer containing 50 mM HEPES, pH 7.5, 50 mM NaCl, 1 mM EDTA, 1% Triton X-100 and protease inhibitor cocktail (cat. no. 04693124001, Roche Diagnostics, Indianapolis, IN). The cell lysates were clarified by a centrifugation at 20,800×g for 10 min at 4°C. For SDS-PAGE of cell lysates, protein concentration was determined with a Bio-Rad protein assay reagent (Bio-Rad Laboratories, Hercules, CA), and 30 µg protein was loaded per lane. Depending on size of protein a 4–20% or 10% SDS-polyacrylamide Tris-HCl gel (Criterion Precast Gel; Bio-Rad Laboratories) was used. Proteins were transferred to Immobilon-P membranes (Millipore) that were blocked with 5% nonfat dried milk dissolved in TTBS (20 mM Tris-HCl, pH 8.0, 0.05% Tween 20, 150 mM NaCl), incubated with primary antibody overnight at 4°C, and subsequently incubated with horseradish peroxidase-labeled secondary antibody. The proteins were visualized with SuperSignal Pico or Femto chemiluminescent reagents (Thermo Scientific) using BioMax films (Eastman Kodak Co., Rochester, NY). For detection of soluble β-catenin, a glutathione S-transferase/E-cadherin pull-down protocol was followed as previously described [77].

## Supporting Information

Table S1 Gene enrichment within gene ontology classifications following treatment of C57MG cells with Rspo2 and Wnt3a.
(XLS)

Table S2 qRT-PCR analysis of gene expression in C57MG cells treated with Rspo2 and Wnt3a alone or combined.
(XLS)

Table S3 Dkk1 inhibition of gene expression in C57MG cells treated with Rspo2 and Wnt3a.
(XLS)

Table S4 siRNA regulation of gene expression in C57MG cells treated with Rspo2 and Wnt3a or BSA alone.
(XLS)

Table S5 Sequences of primers used in RT-PCR analysis.
(DOC)
