# Supplementary material for: Recombinant R-spondin2 and Wnt3a Up- and Down-Regulate Novel Target Genes in C57MG Mouse Mammary Epithelial Cells
Source: PLoS One. 2012 Jan 4;7(1):e29455. doi: 10.1371/journal.pone.0029455 (PMC3251591; doi:10.1371/journal.pone.0029455)
Supplement: Table S5 — Sequences of primers used in RT-PCR analysis. (DOC) [file pone.0029455.s005.doc]

**Table S5.** Sequences of primers used in RT-PCR analysis

| **Primer** | **Sequence** |
| --- | --- |
| 18S-RNA_F | ACC GCG GTT CTA TTT TGT TG |
| 18S-RNA_R | AGT CGG CAT CGT TTA TGG TC |
| Ahr_F | AGC AGC TGT GTC AGA TGG TG |
| Ahr_R | TTC TGG AAG CAG AGG CTG AT |
| Angpt1_F | AGG CTT GGT TTC TCG TCA GA |
| Angpt1_R | TCT GCA CAG TCT CGA AAT GG |
| Angptl4_F | TAG AGT CCC TGA AGG CCA GA |
| Angptl4_R | AAT GAG CTG GGT CAT CTT GG |
| Axin2_F | CAT TTT GGA CGA CCA CCT CT |
| Axin2_R | TTT TGG CAA GGT ACC ACC TC |
| C3_F | AAT AGC CTT CCT GGG AGC AT |
| C3_R | GGA TGT GGC CTC TAC GTT GT |
| CCL5_F | CCC TCA CCA TCA TCC TCA CT |
| CCL5_R | CCT TCG AGT GAC AAA CAC GA |
| CCL7_F | AAT GCA TCC ACA TGC TGC TA |
| CCL7_R | CTT TGG AGT TGG GGT TTT CA |
| Ccnd1_F | AGC AGA AGT GCG AAG AGG AG |
| Ccnd1_R | TGG AAA GAA AGT GCG TTG TG |
| Chrna1_F | CCG TGT TCC TTC TGG TCA TT |
| Chrna1_R | AGA CTC CGA GGA GGA TGT GA |
| Ctgf_F | CAC TCT GCC AGT GGA GTT CA |
| Ctgf_R | AGA TGT CAT TGT CCC CAG GA |
| CXCR6_F | TAG TGG CTG TGT TCC TGC TG |
| CXCR6_R | GGC AGC CGA TAT CCT TCA TA |
| Edn1_F | CTG CCA AGC AGG AAA AGA AC |
| Edn1_R | TTG TGC GTC AAC TTC TGG TC |
| Efnb2_F | AGG AAT CAC GGT CCA ACA AG |
| Efnb2_R | AGA ACC TGG ATT TGG CTT CA |
| Enpp2_F | TCT AGC ATC CCA GAG CAC CT |
| Enpp2_R | CGT TTG AAG GCA GGG TAC AT |
| FGF21_F | CTG GGG GTC TAC CAA GCA TA |
| FGF21_R | CAC CCA GGA TTT GAA TGA CC |
| FGFR5_F | TGT GAA CAC AAC GGT GGA CT |
| FGFR5_R | TTG AGG TAG GAG CCA TCA GG |
| FHL1_F | CTG CGT GGA TTG CTA CAA GA |
| FHL1_R | GTG CCA GGA TTG TCC TTC AT |
| HCK_F | GCC TCA AAA ACA GAG CCA AG |
| HCK_R | GTA CAG TGC GAC CAC AAT GG |
| Helt_F | AAT TAA CCG CTG CTT GAA CG |
| Helt_R | CTC CAC GGT GGT GAG GTA GT |
| Hp_F | TTG AAC GAG CAC ACC TTC TG |
| Hp_R | TCC TGA ACC CAG TCC TTC AG |
| IGF1_F | TGG ATG CTC TTC AGT TCG TG |
| IGF1_R | GTC TTG GGC ATG TCA GTG TG |
| IGFbp2_F | ATG AAG GAG CTG GCT GTG TT |
| IGFbp2_R | CAC CCA GGA TTT GAA TGA CC |
| IL33_F | GCT GCG TCT GTT GAC ACA TT |
| IL33_R | TGA TTG ACT TGC AGG ACA GG |
| InhbA_F | TGG ATG GAG ATG GGA AGA AG |
| InhbA_R | GTC ATT CCA GCC AAT GTC CT |
| Insc_F | CCA GCA CCT CAC TAG CTT CC |
| Insc_R | CCA CTC TCT GCT TGT CAC CA |
| IRF7_F | CCA GTT GAT CCG CAT AAG GT |
| IRF7_R | GAG CCC AGC ATT TTC TCT TG |
| IRS1_F | CAG TAT GGT GGG TGG GAA AC |
| IRS1_R | TAG AAG AAG GCA TGG GGA TG |
| Itgbl1_F | ATG GCG ATA AAT GCG AGT TC |
| Itgbl1_R | TAC ACT GCC CAT GAC CTG AA |
| Klf5_F | ACG TAC ACC ATG CCA AGT CA |
| Klf5_R | GTG GGA GAG TTG GCG AAT TA |
| Klf15_F | TCA TGG AGG AGA GCC TCT GT |
| Klf15_R | TCT CCC AGC AGA CTC TGG AT |
| Klhl30_F | ACA GCG CTG AAT GGA GAG AT |
| Klhl30_R | TGT ACT TGC AGG CAC TGG AG |
| Lcn2_F | GCC CAG GAC TCA ACT CAG AA |
| Lcn2_R | CTG ACC AGG ATG GAG GTG AC |
| Msln_F | GCT TGT GCC CAC TTC TTC TC |
| Msln_R | GGG TGT ATG ACG GTC AGC TT |
| Nanog_F | CCA GTG GAG TAT CCC AGC AT |
| Nanog_R | GAA GTT ATG GAG CGG AGC AG |
| PpFibp2_F | GCC AAG CAA GAA GAA ACG TC |
| PpFibp2_R | GCA GTT GGG GTT GAA CTT GT |
| RhoU_F | CAG AGA TTC GAC GTC ACT GC |
| RhoU_R | GCG TCG AAA ACC TCT TTG AG |
| Rnd3_F | GCA AAT ATG GCC AAG CAG AT |
| Rnd3_R | CTC TTC GCT TTG TCC TTT CG |
| Saa3_F | ACA TGT GGC GAG CCT ACT CT |
| Saa3_R | ATT GGC AAA CTG GTC AGC TC |
| Smoc1_F | CCA CCA ATC CAC AGG CTA CT |
| Smoc1_R | CCT GAA CCA TGT CTG TGG TG |
| Sox9_F | CGA CTA CGC TGA CCA TCA GA |
| Sox9_R | AGA CTG GTT GTT CCC AGT GC |
| Spry1_F | GCT GGT GGA AGA CTT GAA GG |
| Spry1_R | CAT CAT CAT TGG AGC AGT GG |
| STAT1_F | TGG TGA AAT TGC AAG AGC TG |
| STAT1_R | CAG ACT TCC GTT GGT GGA TT |
| TCF4_F | CAC CCC AAG ACC CTT ACA GA |
| TCF4_R | GGT CAG GTC CTC ATC ATC GT |
| Tec_F | AAA ACT AGC ACC CGG ATG TG |
| Tec_R | ATG CCT GAA ACC TGA TGA GC |
| TGFb2_F | CCG GAG GTG ATT TCC ATC TA |
| TGFb2_R | GCG GAC GAT TCT GAA GTA GG |
| TGFbR2_F | CTG TGC AAG TTT TGC GAT GT |
| TGFbR2_R | GGC ATC TTC CAG AGT GAA GC |
| Tspan18_F | GCC ACC TGG AAT TCA GTC AT |
| Tspan18_R | GGT TTC GAA GGT GTT GAG GA |
| Twist1_F | ACG ACA GCC TGA GCA ACA G |
| Twist1_R | CAT CTT GGA GTC CAG CTC GT |
| Wnt9a_F | CGA GTG GAC TTC CAC AAC AA |
| Wnt9a_R | TGG CTT CAT TGG TAG TGC TG |
